# Medical Legislation

**Published:** 1887-07

**Authors:** 


### MEDICAL LEGISLATION.

### THE ILLINOIS AMENDED PRACTICE ACT.

FOLLOWING is the new medical practice act passed by the General Assembly of Illinois at its recent session, and which went into effect July 1st:

SECTION 1. *Be it enacted by the People of the State of Illinois, represented in the General Assembly*, That no person shall practice medicine in any of its departments in this State unless such person possesses the qualifications required by this act. If a graduate in medicine, he shall present his diploma to the State Board of Health fôr verification as to its genuineness. If the diploma is found genuine, and from a legally chartered medical institution in good standing, and if the person named therein be the person claiming and presenting the same, the State Board of Health shall issue its certificate to that effect signed by all of the members thereof, and such certificate shall be conclusive as to the right of the lawful holder of the same to practice medicine in this State. If not a graduate, the person practicing medicine in this State shall present himself before said board and submit himself to such examination as the board may require, and if the examination be satisfactory to the board, the said board shall issue its certificate in accordance with the facts, and the lawful holder of such certificate shall be entitled to all the rights and privileges herein mentioned.

SEC. 2. The State Board of Health shall organize within three months after the passage of this act, it shall procure a seal, and shall receive through its secretary, applications for certificates and examinations; the president and secretary shall have authority to administer oaths, and the board to take testimony in all matters relating to its duties: it shall issue certificates to all who furnish satisfactory proof of having received diplomas or licenses from legally chartered medical institutions in good standing as may be determined by the board; it shall prepare three forms of certificates, one for persons in possession of such diplomas or licenses, the second for candidates examined and favorably passed on by the board, and a third for persons to whom certificates may be issued as hereinafter provided in section 12 of this act; it shall furnish to the county clerks of the several counties a list of all persons receiving certificates. In selecting places to hold its meetings, it shall, as far as is reasonable, accommodate applicants residing in different sections of the State, and due notice shall be published of all its meetings for examination. Certificates shall be signed by all the members of the board, and the secretary of the board shall receive from the applicant a fee of five (5) dollars for each certificate issued to such graduate or licentiate. Graduates or licentiates in midwifery to pay the sum of two (2) dollars for each certificate. All such fees for cèrtificates shall be paid by the secretary into the treasury of the board.

SEC. 3. The verification of the diploma shall consist in the affidavit of the holder and applicant that he is the lawful possessor of the same, and that he is the person therein named. Such affidavit may be taken before any person authorized to administer oaths, and the same shall be attested under the hand and official seal of such officer, if he have a seal; and any person swearing falsely shall be deemed guilty of perjury, and punished accordingly. Graduates may present their diplomas and affidavits as provided in this act, by letter or by proxy, and the State Board of Health shall issue its certificate the same as though the owner of the diploma was present.

SEC. 4. All examinations of persons not graduates or licentiates, shall be made directly by the board, and the certificates given by the board shall authorize the possessor to practice medicine and surgery in the State of Illinois.

SEC. 5. Every person holding a certificate from the State Board of Health shall have it recorded in the office of the clerk of the county in which he resides within three months from its date, and the date of recording shall be indorsed thereon. Until such certificate is recorded as herein provided the holder thereof shall not exercise any of the rights or privileges conferred therein to practice medicine. Any person removing to another county to practice shall record the certificate in like manner in the county to which he removes, and the holder of the certificate shall pay to the county clerk the usual fees for making the record.

SEC. 6. The county clerk shall keep, in a book provided for the pnrpose, a complete list of the certificates recorded by him, with the date of the issue of the certificate. If the certificate be based on a diploma or license, he shall record the name of the medical institution conferring it, and the date when conferred. The register of the county clerk shall be open to public inspection during business hours.

SEC. 7. The fees for the examination of non-graduates shall be as follows: Twenty (20) dollars for an examination in medicine and surgery; ten (10) dollars for an examination in midwifery only; and said fees shall be paid into the treasury of the board. If an applicant fails to pass said examination his or her fee shall be returned. Upon successfully passing the examination the certificate of the board shall be issued to the applicant without further charge.

SEC. 8. Examinations may be made in whole or in part in writing, and shall be of an elementary and practical character, but sufficiently strict to test the qualifications of the candidate as a practitioner.

SEC. 9. The State Board of Health may refuse to issue the certificates provided for in section 2 to individuals guilty of unprofessional or dishonorable conduct, and may revoke such certificates for like causes. In all cases of refusal or revocation the applicant may appeal to the Governor, who may affirm or overrule the decision of the board, and this decision shall be final.

SEC. 10. Any person shall be regarded as practicing medicine, within the meaning of this act, who shall treat, operate on, or prescribe for any physical ailment of another. But nothing in this act shall be construed to prohibit service in cases of emergency, or the domestic administration of family remedies. And this act shall not apply to commissioned surgeons of the United States Army, Navy or Marine Hospital Service in the discharge of their official duties.

SEC. 11. Any itinerant vendor of any drug, nostrum, ointment or appliance of any kind, intended for the treatment of disease or injury, or who shall, by writing or printing or any other method, profess to cure or treat any disease or deformity, by any drug, nostrum, manipulation or other expedient, shall pay a license of one hundred (100) dollars per month into the treasury of the board, to be collected by the State Board of Health, in the name of the People of the State of Illinois for the use of said Board of Health. And it shall be lawful for the State Board of Health to issue such license on application made to the State Board of Health, such license to be signed by the President of the Board, and attested by the secretary of the board, with the seal of the board. Any such itinerant vendor who shall vend or sell any such drug, nostrum, ointment or appliance without having a license so to do, shall, if found guilty, be fined in any sum not less than one hundred dollars, and not exceeding two hundred dollars for each offense, to be recovered in an action of debt before any court of competent jurisdiction. But such board may for sufficient cause refuse such license.

SEC. 12. Any person practicing medicine or surgery in the State without the certificate issued by this board in compliance with the provisions of this act, shall for each and every instance of such practice forfeit and pay to the people of the State of Illinois for the use of the said State Board of Health the sum of one hundred (100) dollars for the first offense, and two hundred (200) dollars for each subsequent offense, the same to be recovered in an action of debt before any court of competent jurisdiction, and any person filing or attempting to file as his own the diploma or certificate of another, or a forged affidavit of identification, shall be guilty of a felony, and upon conviction, shall be subject to such fine and imprisonment as are made and provided by the statutes of the State for the crime of forgery: *Provided:* that all persons who have been practicing medicine continuously for ten years within this State prior to the taking effect of the act to which this is an amendment, and who have not under said original act obtained a certificate from said Board of Health to practice medicine in this State, shall upon proper application to said Board of Health receive such certificate, unless it shall be ascertained and determined by said Board of Health that the person so applying for a certificate is of immoral character, or guilty of unprofessional or dishonorable conduct, in which case, said Board of Health may reject such application: *And, provided,* that such application for a certificate shall be made within six months after the taking effect of this act; and all persons holding a certificate on account of ten years' practice shall be subject to all the requirements and discipline of this act, and to the act to which this is an amendment, in regard to their future conduct in the practice of medicine the same as all other persons holding certificates, and all persons not having applied for or received such certificate within six months after the taking effect of this act, and all persons whose applications have for the causes herein named been rejected or certificates revoked, shall, if they shall practice medicine, be deemed guilty of practicing in violation of law, and shall suffer the penalties herein provided.

SEC. 13. Upon conviction of either of the offenses mentioned in this act, the court shall, as part of the judgment, order that the defendant be committed to the common jail of the county until the fine and costs are paid, and upon failure to pay the same immediately the defendant shall be committed under said order. *Provided,* that either party may appeal in the same time and manner as appeals may be taken in other cases, except that where an appeal is prayed in behalf of the people, no bond shall be required to be filed, whether the appeals be from a justice of the peace, or from the county or circuit court, or from the appellate court. But it shall be sufficient, in behalf of the people of the State of Illinois, for the use of the State Board of Health, to pray an appeal, and thereupon an appeal may be had without bond or security.

SEC. 14. All acts and parts of acts inconsistent or in conflict with this act are hereby repealed.

